# A Case Report of Severe Factor XI Deficiency during Cardiac Surgery: Less Can Be More

**DOI:** 10.3390/jcdd9040118

**Published:** 2022-04-15

**Authors:** Toshinobu Kazui, Vance G. Nielsen, Spencer D. Audie, Rajagopalan M. Venkataramani, John T. Bryant, Kristin Swenson, Paul M. Ford

**Affiliations:** 1 Department of Surgery, The University of Arizona College of Medicine, Tucson, AZ 85724, USA; tkazui@surgery.arizona.edu; 2 Department of Anesthesiology, The University of Arizona College of Medicine, Tucson, AZ 85724, USA; saudie@anesth.arizona.edu (S.D.A.); swensonk@email.arizona.edu (K.S.); pford@anesth.arizona.edu (P.M.F.); 3 Perfusion Department, Banner University Medical Center, Tucson, AZ 85719, USA; rajagopalan.venkataramani@bannerhealth.com; 4 Department of Anesthesiology, Veterans Administration Hospital, Tucson, AZ 85723, USA; jackaldoc@gmail.com

**Keywords:** cardiopulmonary bypass, severe factor XI deficiency, perioperative outcomes, rotational thromboelastimetry

## Abstract

Severe congenital Factor XI (FXI) deficiency (<20% normal activity) can be associated with significant bleeding disorders, and there has been great concern for severe bleeding following cardiac surgery requiring cardiopulmonary bypass (CPB) in this patient population. Over the past four decades remarkably different approaches to this problem have been taken, including the administration of blood volumes of fresh frozen plasma, administration of activated recombinant Factor VII, and diminutive administration of heparin. We describe a case wherein the patient was assessed in the perioperative period with a point-of-care, viscoelastic hemostasis device (ROTEM), with changes in the intrinsic/Factor XII-dependent coagulation pathway determined before, during, and after CPB. Fresh frozen plasma was administered in small amounts (5–7.5 mL/kg) just before surgery began and just before cessation of CPB. Administering fresh frozen plasma to the patient to nearly normalize in vitro ROTEM hemostasis values at times when hemostasis was needed resulted in no important bleeding occurring or need of further transfusion of other blood products. In conclusion, by using small amounts of fresh frozen plasma guided by ROTEM, an evidenced-based, precision medicine approach resulted in optimized patient care and outcome.

## 1. Introduction

Coagulopathy following surgical procedures requiring cardiopulmonary bypass (CPB) is a multifactorial problem that can involve cellular and plasmatic components of the circulation [1]. The severity of coagulopathy after CPB is dependent on several factors, including hemodilution and time of perfusion–which consumes, via activation of the contact protein pathway (Factor XII-dependent), multiple key serine proteases that comprise the pathway, in addition to platelets and fibrinogen, depending on the degree of heparin-mediated anticoagulation [1]. Factor XI (FXI) is a serine protease within the contact protein pathway, and it serves as the most proximate enzyme that permits amplification of thrombin generation via its activation by thrombin generated by the tissue factor pathway [2]. FXI deficiency that is severe is defined as an activity <20% of normal, and the incidence of the mostly autosomal-recessive disorder is approximately one in a million among the general population [2]. Of interest, bleeding tendency in the presence of severe FXI deficiency can be quite variable, with typical treatment consisting of fresh frozen plasma (FFP) [2]. Taken as a whole, patients presenting for procedures requiring CPB with coexisting severe FXI deficiency could be at significant risk for unpredictable degrees of bleeding in the postoperative period.

A brief review of the literature concerning such patients reveals remarkably different degrees of intervention, varying from simply using less heparin for CPB to days of plasmapheresis with FFP prior to operation [3,4,5,6,7,8,9,10,11,12,13]. We present a patient with severe FXI deficiency that required coronary artery bypass grafting (CABG) and CPB that was treated with incremental administrations of FFP in the intraoperative period coupled with hemostatic assessments with rotational thromboelastimetry (ROTEM) to guide therapy.

## 2. Case Report

The patient was a 76-year-old, 98 kg male with a recent history of dyspnea on exertion found to have the following coronary artery stenoses: left anterior descending 70–80%; obtuse marginal 70%; left circumflex, ramus 70%; and posterior descending artery 60–70%. His left ventricular ejection fraction was 58%. He also had a history of severe FXI deficiency that was discovered 12 years prior before having a lumbar laminectomy. He was treated with FFP prior to that surgery but received no blood products afterward. His history prior to that surgery included a life-long lack of bleeding events and no problems with bleeding after dental procedures.

The hematology service assessed our patient and found that he had normal standard hemostatic assessment values with the exception of a prolonged activated prothrombin time (aPTT) as obtained by our central laboratory which is displayed in Table 1 in the Pre-Op time period. He was subsequently determined to have a FXI activity that was 2% of normal, indicative of severe deficiency [2]. The hematology service recommended the administration of 10 mL/kg of FFP prior to surgery and that antifibrinolytic medications be used intraoperatively.

On the day of surgery, a number of hemostatic assessments were made as indicated in Table 1. The data were obtained shortly after an uneventful anesthetic induction and placement of invasive monitors and are displayed in the Pre-Op time period. With regard to our routine viscoelastic assessments with a ROTEM Delta system (International Laboratory, Bedford, MA, USA), there was prolongation of the clotting time (CT) value and depression of α value compared to the normal range in the InTem sample, which is activated via the contact protein pathway (e.g., intrinsic pathway, FXII activated by ellagic acid). In contrast, the CT of the ExTem, a tissue factor-activated (e.g., extrinsic pathway, Factor VII-dependent) test, was within normal limits. Lastly, the maximum clot firmness is all three ROTEM tests were within the normal range, which included the FibTem, a tissue-factor activated, platelet-inhibited sample.

Just prior to commencement of surgery, the patient had 2 units of FFP (500 mL) administered, or approximately half of the dose recommended by the hematology service, with another set of ROTEM tests performed shortly after administration. While the CT value of the InTem did not enter the normal range, it did decrease by 117 s, and the α value entered the normal range. The intravenous administration of the antifibrinolytic agent, aminocaproic acid, was also commenced as part of our routine clinical practice (5 gm loading dose followed by a 20 mg/kg/h infusion). The rationale for this approach was that the perioperative team wished to observe the clinical response in terms of bleeding and correlate it with the laboratory results. There was no inordinate bleeding observed with surgical dissection. The activated clotting time (ACT) value of the blood sample obtained after FFP administration determined with a Medtronic HMS Plus Hemostasis Management System (Medtronic, Inc., Tempe, AZ, USA) was prolonged beyond the range of normal by only 18 s. Further, there was normalization of CT and α values in the InTem sample, indicative of increased FXI activity after FFP administration. After surgical preparation was complete, 15,000 IU of unfractionated heparin was administered, with a resultant ACT value of 645 s observed. The dosage of heparin was determined via an in vitro heparin concentration-response relationship generated by the Medtronic system so as to have an ACT value > 480 s prior to CPB. CPB was then commenced.

CABG × 4 (left internal mammary artery to left anterior descending artery; saphenous vein grafts to diagonal and obtuse marginal arteries; in situ right internal mammary to ramus) was performed with an aortic cross clamp time of 63 min and a CPB perfusion time of 87 min. After an hour of perfusion, a blood sample was analyzed with a HepTem test, which is the same as an InTem, but with the addition of heparinase to digest heparin so as to be able to assess coagulation. As seen in Table 1 in the CPB time period, coagulation had deteriorated compared to the InTem sample prior to CPB. Given the prolongation of the CT value and depression of the α value of the HepTem sample during CPB, an additional 3 units of FFP (750 mL) was added to the CPB pump over 10 min just prior to separation from CPB. The patient was successfully separated from CPB, and heparin anticoagulation was neutralized with 150 mg of protamine.

Decannulation and chest closure was uneventful, with no meaningful bleeding noted. As displayed in the Post-CPB period, the ACT value was within normal limits, and InTem CT and α values were similar to that observed after the first administration of FFP prior to CPB. Further, upon arrival to the cardiac intensive care unit, the aPTT, PT, and fibrinogen concentration values were within normal limits. While the platelet count was small compared to the normal range, given the normal MCF values observed in all three ROTEM tests and lack of clinical bleeding, no platelets or other blood products were transfused. The following day the FXI activity was found to be 17% and, given the 50–70-h half-life of FXI [2], it was likely that this activity was >20% the day before after FFP administration, rendering the patient no longer severely deficient. The patient had an uncomplicated postoperative course and was discharged home 6 days after operation.

## 3. Discussion

This case report should serve as an evidence-based approach to the management of hemostasis of a patient afflicted with severe FXI-deficiency requiring CPB. Rather than simply administering 10 mL/kg of FFP at the beginning of surgery, the authors chose to assess hemostasis with an intraoperative, point-of-care (POC) test that should have detected deficiencies in the contact protein pathway (InTem). Then, 5 mL/kg of FFP was administered, with an improvement in InTem parameters noted, which correlated with the clinical assessment of minimal bleeding with surgery prior to CPB. Subsequently, using the HepTem test during CPB, it was discerned that the contact protein pathway had been compromised most likely secondary to consumption of coagulation enzymes and hemodilution [1]. This allowed for timely administration of another 7.5 mL/kg of FFP just prior to separation from CPB. The additional 2.5 mL/kg of FFP was administered to account for changes in circulating volume secondary to the CPB pump volume. The subsequently collected blood samples after protamine administration provided hemostatic assessment parameter values that were fairly normal, which, again, was consistent with the clinical situation wherein post-CPB bleeding was not remarkable. In summary, by assessing hemostatic competency with the appropriate POC tests coupled with the judicious administration of FFP just prior to the time such hemostasis was needed in vivo resulted in an excellent clinical outcome. This approach will now be contrasted with the management of previous patients afflicted with severe FXI deficiency as summarized in Table 2.

As displayed in Table 2, there has been extraordinary variation in approach to the management of patients with severe FXI deficiency undergoing cardiac surgical procedures. In general, the administration of FFP or FXI concentrate was designed to obtain a more normal aPTT value or >20% FXI activity [3,4,5,6,7,9,10,12,13], whereas administration of rFVIIa was designed to generate thrombin independent of the contact protein pathway without regard to aPTT or FXI activity to effect hemostasis in the perioperative period [8,11]. However, the strategies employed in these cases often involved blood volume to multiples of blood volumes of FFP before, during and after surgery [3,4,5,7,12,13], which was likely unnecessary given the half-life of FXI. One case required blood product replacement equivalent to 55 L [5], which was not likely related to FXI activity as opposed to surgical misadventure. Further, the case requiring nine administrations of rFVIIa (90 µg/kg) did not document bleeding or other blood products during the day of operation [8], but reported only 50 mL blood loss on the first postoperative day. The impact of FXI activity on coagulation was likely minimal given the extraordinary utilization of rFVIIa and postoperative cardiovascular and neurological events [8]. Another unusual approach was the administration of only a small dose of heparin (8000 U) to anticoagulate a patient for CPB, as the ACT value was no longer measurable [9]. The FXII-dependent pathway is only one source of thrombin generation during CPB, with the FVII-dependent pathway and activated platelets being other sources of thrombin generation in this setting [14], making thrombus in the CPB pump still possible; thus, typical doses of heparin to anticoagulate such patients is still recommended, with high-dose thrombin time used to assess anticoagulation as this test does not depend on the activity of the FXII-dependent pathway [15]. When considered in conglomerate [3,4,5,6,7,8,9,10,11,12,13], these cases did not use a precision medicine approach to administer FFP (or any other agent) to achieve laboratory-based values incrementally to prevent bleeding in the two important times during cardiac surgery, before and after CPB.

When considering the biochemistry of therapeutic approach, the use of FXI concentrate or FFP seems the most facile. Potential disadvantages of using FFP include fluid overload and dilution of platelets, causing adverse hemodynamics/pulmonary status and necessitating platelet transfusion, respectively. In our circumstance, the FFP was administered in divided doses, with the first dose followed by surgical loss and the second during CPB when hemofiltration is routinely used to remove excess fluid from the patient and circuit. Further, our patient was monitored with a pulmonary artery catheter and transesophageal echocardiography probe, allowing appropriate fluid management to optimize hemodynamics. It should also be noted that CPB results in consumption of not just FXI, but also FXII, Factor IX, and Factor X, which are all replaced by FFP. Thus, FXI concentrate is most useful prior to CPB, but a second administration after CPB may not compensate for the loss of other contact pathway enzymes in the establishment of post-surgical hemostasis. FXI is also not universally available, and its administration has been associated with significant and even fatal thrombosis [16]. Further, the use of antifibrinolytics, such as aminocaproic acid, is not recommended when administering FXI concentrate as the risk of thrombosis is enhanced [16]. This is problematic, as the use of antifibrinolytics to preserve platelet number and function is an established practice in cardiac surgery. The administration of FFP also includes endogenous anticoagulants, such as antithrombin and protein C, which may compensate for any tendency towards hypercoagulation when exogenous FXI is introduced into a patient with severe FXI deficiency–a benefit not enjoyed when administering FXI concentrate. In summary, both clinical approaches have their benefits and risks, and the therapy chosen should be done so carefully with consultation with appropriate hematology specialists.

As a practical matter, patients living long enough to acquire heart disease requiring CPB for cardiac surgery serve as a testament to the concept that a patient’s clinical bleeding and outcome should not be predicted by the activity of any one enzyme, such as FXI. This is most likely true for those patients found in later life to have severe FXI deficiency, which is indicative of other potential compensatory mechanisms that maintain day-to-day hemostasis (e.g., platelet reactivity or other coagulation enzyme/protein concentrations). However, as previous case reports have demonstrated over a 4-decade period [3,4,5,6,7,8,9,10,11,12,13], such a practical assessment of patient circumstance with evidence-based hemostatic management has not been the rule. Therefore, this case report and narrative review may serve as evidence of the old edict that “less can be more”; put another way, perhaps interventions (e.g., multiple liters of FFP) designed to maintain a large FXI activity for a prolonged period of time based on aPTT or FXI activity determination could be replaced with an evidence-based, precision medicine approach, guided with point-of-care hemostatic assessments to optimize outcomes with a far smaller magnitude of blood product/clotting factor administration.

## Figures and Tables

**Table 1:** Perioperative hemostatic assessments.

Parameter	Pre-Op	Pre-CPB	CPB	Post-CPB
aPTT (s, 24.0–36.5)	68.6	-	-	35.1
INR (0.9–1.1)	1.0	-	-	1.0
Fibrinogen (mg/dL, 200–465)	-	-	-	290
Platelet Count (1000/µL, 130–450)	206	-	-	114
ACT (s, 100–140)	-	158	525–665	125
InTem				
CT (s, 122–208)	397	280	366 *	256
α (°, 70–81)	67	75	67 *	69
MCF (mm, 51–72)	57	64	56 *	57
ExTem				
CT (s, 43–82)	77	65	-	85
MCF (mm, 52–70)	59	64	-	60
FibTem				
MCF (mm, 7–24)	17	17	-	15

Parameters: ACT = activated clotting time; aPTT = activated partial thromboplastin time; α = angle, a measure of the velocity of thrombus formation; CT = clotting time, a measure of the time to onset of coagulation; INR = international normalized ratio; and MCF = maximum clot firmness, a measure of clot strength. ROTEM test types: ExTem = coagulation activated via tissue factor mediated factor VII-dependent pathway; FibTem = ExTem with platelet-inactivation; InTem = coagulation activated via the factor XII-dependent, contact protein pathway; and * InTem with heparinase to digest heparin, also known as HepTem. Ranges of normal in parentheses were generated within the institution by the clinical laboratory under the supervision of the Department of Pathology. Time Periods: Pre-Op = values obtained prior to treatment with FFP; Pre-CPB = values obtained after the first FFP administration before CPB commenced; CPB = samples obtained during CPB but before the second administration of FFP; and Post-CPB = samples obtained after the second administration of FFP following the cessation of CPB.

**Table 2:** Management of severe FXI deficiency for CPB and outcomes.

Reference	Procedure	FXI Activity	Intervention	Outcome
[3]	CABG	14%	FFP (7 L) before, during and after operation for 2 days.	Normal operation and recovery.
[4]	CABG	<1%	No FXI inhibitor present. Daily plasmapheresis for 2 days prior to operation (6.1 L FFP); FFP (800 mL), platelets, RBC transfusions during operation. Platelets, cryoprecipitate and FFP (400 mL) in intensive care unit.	Blood loss 850 mL in first postoperative hour; 950 mL over 12 h.
[5]	CABG	8%	Heat-treated FXI concentrate preoperatively (FXI increased to 125% normal). Multiple units of FFP, whole blood, platelets and cryoprecipitate during and after operation.	Blood loss 55 L over 48 h after operation.
[6]	CABG	4.5%	FFP (8 U) before and during operation. Four units of RBC after operation.	Normal postoperative course.
[7]	Repeat AVR	<1%	FXI inhibitor present. Daily plasmapheresis for 3 days prior to operation with FFP (7.6 L). Daily plasmapheresis for 4 days after operation with FFP (18.8 L).	Required mediastinal exploration for tamponade on day of operation.
[8]	Mitral Valvuloplasty	5%	Administered rFVIIa (90 µg/kg) administered after CPB. Repeated every 2–4 h after operation for a total of 8 more administrations.	Postoperative period complicated by cardiac arrest on day 2 followed by 2 weeks of neurological abnormalities.
[9]	AVR	5%	Administered only 8000 U of heparin for CPB. FFP (4 U) and tranexamic acid (3 gm) administered after CPB. FFP (2 U) administered on first postoperative day.	Four units of RBC were needed during CPB. Total blood loss was 980 mL and postoperative course was uneventful.
[10]	AVR	2.9%	Administered FXI concentrate (15 U/kg) prior to surgery; tranexamic acid (5 gm) during operation; FFP (4 U) after CPB. FXI concentrate (1000 U) administered on third postoperative day.	Patient bled 720 mL over three days. Normal postoperative course.
[11]	AVR	<1%	FXI inhibitor present. Administered tranexamic acid (15 mg/kg bolus and 4.5 mg/kg/h) during operation, administered rFVIIa (15 µg/kg) after CPB. Tranexamic acid (oral, 800 mg three times a day) for three days after operation.	No bleeding or administration of blood products. Normal postoperative course.
[12]	Repeat Aortic Root Replacement	<1%	Administered FFP (15 U) over two days before operation; aminocaproic acid (10 gm bolus, 2 gm/h infusion) during operation and for the next three days; FFP (8 U) just before CPB stopped, then FFP (2 U) in operating room and FFP (7 U) in ICU.	Sternum left open on day of surgery, closed the next day. Uncomplicated postoperative course.
[13]	CABG	11.4%	Administered FFP (4U) 6 days prior, FFP (4 U) one day prior, FFP (6 U) intraoperatively and FFP (2 U) first postoperative day.	No bleeding, platelet concentrate (1 U) and RBC (1 U) administered. Normal postoperative course.

AVR = aortic valve replacement; CABG = coronary artery bypass grafting; CPB = cardiopulmonary bypass; FFP = fresh frozen plasma; ICU = intensive care unit; rFVIIa = activated recombinant factor VII.
